# Structural and enzymatic characterisation of the Type III effector NopAA (=GunA) from *Sinorhizobium fredii* USDA257 reveals a Xyloglucan hydrolase activity

**DOI:** 10.1038/s41598-020-67069-4

**Published:** 2020-06-18

**Authors:** Jonathan Dorival, Sonia Philys, Elisa Giuntini, Romain Brailly, Jérôme de Ruyck, Mirjam Czjzek, Emanuele Biondi, Coralie Bompard

**Affiliations:** 1 Sorbonne Université, CNRS, Integrative Biology of Marine Models (LBI2M), Station Biologique de Roscoff (SBR), 29680 Roscoff, Bretagne France; 2 CNRS, Univ. Lille, Unité de Glycobiologie Structurale et Fonctionnelle, 59000 Lille, France; 3 CNRS, LCB, Aix Marseille University, Marseille, France

**Keywords:** Biochemistry, Biophysics, Microbiology, Plant sciences, Structural biology

## Abstract

Rhizobia are nitrogen-fixing soil bacteria that can infect legume plants to establish root nodules symbiosis. To do that, a complex exchange of molecular signals occurs between plants and bacteria. Among them, rhizobial Nops (Nodulation outer proteins), secreted by a type III secretion system (T3SS) determine the host-specificity for efficient symbiosis with plant roots. Little is known about the molecular function of secreted Nops (also called effectors (T3E)) and their role in the symbiosis process. We performed the structure-function characterization of NopAA, a T3E from *Sinorhizobium fredii* by using a combination of X-ray crystallography, biochemical and biophysical approaches. This work displays for the first time a complete structural and biochemical characterization of a symbiotic T3E. Our results showed that NopAA has a catalytic domain with xyloglucanase activity extended by a N-terminal unfolded secretion domain that allows its secretion. We proposed that these original structural properties combined with the specificity of NopAA toward xyloglucan, a key component of root cell wall which is also secreted by roots in the soil, can give NopAA a strategic position to participate in recognition between bacteria and plant roots and to intervene in nodulation process.

## Introduction

Many bacterial pathogens use type III secretion systems (T3SS) to inject virulence factors, named effectors, directly into the cytoplasm of target eukaryotic cells. Most of the T3SS apparatus components are conserved among plant and animal pathogens, suggesting a common mechanism of recognition and secretion of effectors (for review see^1^). Secretion of effectors depends on the presence of secretion signals composed by 20 to 30 non-conserved amino acids at the N-terminus. Most of T3SS effectors (T3E) are predicted to possess a N-terminal intrinsically disordered (ID) region enriched in serine residues and required for secretion^2^. Many secreted proteins also depend on the interaction with a cytoplasmic specific T3S chaperone^3^ that stabilize ID regions in bacterial cytoplasm prior translocation. For SopB, a T3E of *Salmonella* it has been proposed that its cognate chaperone SigE may be responsible of the formation of ring like hexamers, conformation that may be required for substrate recognition by the type three apparatus^4^. In pathogenic bacteria, T3SS genes coding for the secretion apparatus, effectors and accessory proteins are clustered in pathogenicity islands (PAIs) and organized in operons.

Interestingly, this secretion strategy is also used by non-pathogenic organisms contributing to symbiotic interactions with hosts as shown for rhizobia^5,6^. Rhizobia are soil bacteria having the ability to establish a specific symbiotic association with leguminous host-plant roots. This interaction leads to the formation of root nodules by the plant, specialized organs in which bacteria differentiate into nitrogen fixing bacteroids able to convert atmospheric nitrogen into ammonia for the usage in plant growth^7^. This process starts with the secretion of flavonoids by plant roots that interact with NodD. After interaction with appropriate flavonoids, NodD activates the expression of many symbiotic genes including those involved in the production and secretion of Nod factors^8^. Nod factors are recognized by specific plants and involved in the biogenesis of root-nodules. Plant flavonoids also induce the expression of T3SS genes *via* NodD^9^ and the secretion of T3E also called Nops (Nodulation outer proteins). In certain rhizobia (mainly *Sinorhizobium fredii*, *Mesorhizobium loti* and *Bradyrhizobium species*)^10^, T3E are important for determining host-specificity in rhizobia-legume symbioses. The set of T3E secreted by rhizobia can either affect positively or negatively the mutualistic association depending on both the bacterium and the host plant^11–13^. Very recent results revealed that ErnA, a T3E widely distributed among bradyrhizobia can confer the ability to form nodules in legumes. This effector localizes at the host cell nucleus and together with the action of five other T3Es allows bradyrhizobia to activate the nodulation process in a T3SS-dependent process^14^.

Functional T3SS have been reported in many rhizobial species^6,9,15–17^ (for review^10^). Compared with pathogenic bacteria little is known about the function of rhizobial T3E in the symbiosis process and only few of them have been functionally characterized^11^. In rhizobia genes encoding the T3SS apparatus are clustered in Symbiotic Islands similar to PAIs. However, T3E genes are scattered throughout the genome (for review^18^).

Comparative sequence analysis revealed that about 10 of the 20 components of the T3SS apparatus are conserved among pathogenic bacteria, constituting the core component of the secretion system^1^. Nops have been identified using multidisciplinary approaches. Some of them are involved in the formation of surface bacterial appendages, called type 3 pili, similar to the appendages associated with plant pathogen T3SS and whose structure is adapted to the interaction with plant cell wall^1^. Other Nops are T3E proteins that are recognized and translocated by T3SS into plant cells or secreted. A major difference between pathogenic and symbiotic T3SS is the absence of genes coding for T3S chaperones in rhizobial symbiotic islands.

Nops possess a non-conserved N-terminal 20 to 30 amino acids sequences allowing their secretion by rhizobial T3SS but also through T3SS of *Pseudomonas syringae*, a plant pathogen, showing that N-terminal secretion signals are interchangeable among pathogenic and symbiotic bacteria^19^.

At the early stage of symbiosis and before translocation, certain Nops can be secreted upon flavonoid induction into the symbiotic area or stay in contact with rhizobial appendages^15^. Some of them have been identified as putative cell-wall degrading proteins whose action could facilitate effector translocation^20^. One of them, NopAA (previously named as GunA) is conserved in five *Bradyrhizobium japonicum* strains, as well as in *Sinorhizobium fredii* USDA257 and HH103 with which it shares 100% protein sequence identity^21^ but not in NRG234^19,21^. The N-terminal end of NopAA contains Type III secretion signals and the C-terminal domain of NopAA has been classified in glycoside hydrolase family 12 (GH12) of the CAZY database. Recombinant NopAA from *B. diazoefficiens* USDA 110 was shown to have cellulase activity^22^, although it is not crucial for the nodulation process. In *Sinorhizobium fredii* HH103, GunA has been shown to possess also a cellulase activity but it also plays an important role in host specificity as its inactivation differently affects symbiosis with soybean and with cowpea^23^.

The structure, function and regulation of T3SSs in animal-and phytopathogenic bacteria have been extensively studied in last decades but little is known about T3SSs mechanism and role in rhizobia. In order to decipher the molecular function of this Nop in symbiotic association with host-plant we started structural analysis of NopAA from *Sinorhizobiun fredii* USDA257 (Accession number JX135409), an ortholog of GunA in *B. diazoefficiens* and *Sinorhizobium fredii* HH103. In this study we report the enzymatic characterization and the atomic structure of NopAA, which is the first high resolution structure of a rhizobial T3E. Obtained results showed that NopAA has a xyloglucanase activity that may play a role in cell wall disruption facilitating effector translocation. This work also allows the visualisation, for the first time, of the molecular organization of the active domain together with the secretion domain of a type III effector.

## Results and Discussion

### NopAA C-terminal domain structure displays the typical GH12 family fold

NopAA as all T3Es possesses a secretion region at its N-terminus. We identified this region as the first 48 amino acids of the protein by sequence alignment with the analogous sequences of GH12 enzymes. This region, predicted to be disordered, has been deleted to allow crystallization of the protein. We obtained unique crystals of the truncated effector NopAAΔ48 from *Sinorhizobium fredii* USDA257 in complex with cellobiose, diffracting to 2.2 Å resolution. Crystals belong to the monoclinic space group C2 with cell parameters a = 135.41 Å b = 108.32 Å c = 84.71 Å β = 113.11° (Table 1). The structure was solved using an ensemble of models generated by Phaser (see material & method part). Crystals contain 53% of solvent and four molecules (molecule A, B, C, D) per asymmetric unit covering the entire NopAAΔ48 chain (except molecule B, Supplementary Fig. S1). These molecules are identical for the enzyme core structure but slightly different in the orientation of the long loops surrounding the active cleft (Supplementary Fig. S1). The region 141–146 of the loop β9/β10 is not defined in the electron density for molecule B, which is not complexed with cellobiose (Supplementary Fig. S1).

**Table 1:** Data collection and refinement statistics for NopAAΔ48/cellobiose complex (Values in parentheses are for the highest resolution shell (2.47–2.36 Å)).

Data collection Diffraction source	Id23-2, ESRF
Wavelength (Å)	0.873
Temperature (K)	100
Detector	Pilatus3_2M
Crystal-to-detector distance (mm)	270.47
Oscillation range (°)	0.05
Exposure time (s)	0.04
Total rotation range (°)	180
Space group	C2
Unit cell parameter (Å,°)	a = 135.41 b = 108.32 c = 84.71 β=113.11
Resolution (Å)	44.72–2.26 (2.47–2.36)
R_meas_ %	16.3 (85)
Completeness (%)	99.7 (99.7)
Total No. of reflections	194021 (24853)
No. of unique reflections	46223 (5918)
Multiplicity	4.2 (4.2)
CC1/2 (%)	88.14 (59.9)
〈I/σ(I)〉	9.1 (1.82)
**Refinement**	
Resolution range	44.47–2.2 (2.28–2.2)
Completeness (%)	99.89 (99.93)
R_factor_ (%)	18.07 (26.59)
R_free_ (%)	21.45 (30.23)
No. of protein atoms [average B values (Å^2^)	6821 (47.4)
No. of solvent atoms	341 (45.4)
No. ligand atoms	35 (30.4)
Ramachandran plot (%)	
Favored	97.49
outliers	0.68
RMSD from ideal values	
Bond distances (Å)	0.016
Bond angle (°)	1.19
**Ligand occupancy**	
Glucose (chain A)	0.3
Cellobiose (chain D)	0.3
PDB code	6SDU

**Figure 1:**
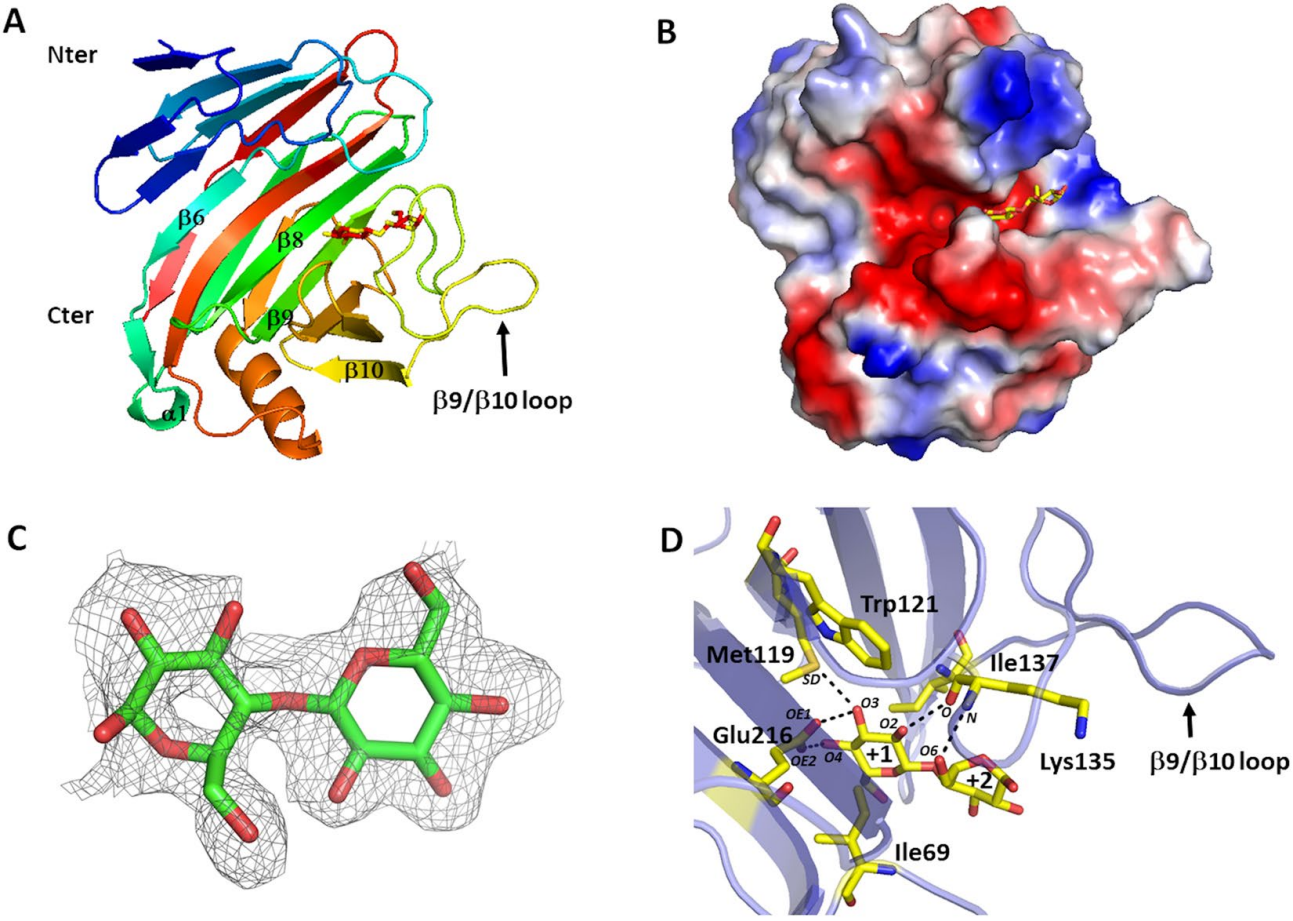
Crystal structure and cellobiose binding. (**A**) Overall structure of NopAA (chain D) in complex with cellobiose. The protein is shown as a cartoon model and is coloured in rainbow from navy blue in the N-terminus to red at the C-terminus. Secondary structure elements discussed in the text are labelled (β for β strands and α for α helix) and numbered starting from N-terminus. Glucose moieties of cellobiose molecule bound in subsites +1 and +2 are shown as balls and sticks, (**B)** NopAA in the same orientation as in panel A is represented in surface potential mode. Surface positively and negatively charged aminoacid residues are colored blue and red respectively. Glucose moieties of cellobiose bound in subsites +1 and +2 are shown as balls and sticks, (**C)** Polder map of cellobiose in chain D contoured at 3.6 σ (**D)** Mode of binding of cellobiose in the active site of NopAA (shown as semi-transparent light violet cartoon). Contacting amino acid residues and cellobiose are shown in balls and sticks. Atoms involved in hydrogen bonds are labelled.

NopAAΔ48 adopts a β-jelly roll fold typical of enzymes of family GH12^24^ with a large substrate binding cleft, containing substrate binding subsites running across the surface of the enzyme (Fig. 1A,B). Structural alignment using DALI server^25^ confirms high similarity with endoglucanases from GH12 family, with a slight advantage (better Z score) for Xyloglucan specific endoglucanase (4NPR, Z score 28.9, rmsd 1.5 Å for 221 matched Cα positions) versus cellulases (1OLR^26^, Z score 27.4, rmsd 1.6 Å for 224 matched Cα positions).

GH12 family enzymes catalyse the reaction with retention of the anomeric configuration and with two glutamic acids (acid/base and nucleophile) as catalytic residues. In NopAA, these conserved residues are Glu117 (nucleophile) and Glu216 (acid/base residue). The electron density shows sugar units in the positive subsites of the molecules D and A of the asymmetric unit: two glucose moieties of the product cellobiose in molecule D (subsites +1 and +2) and one moiety (subsite +1) in molecule A could be refined in the crystal structure with an occupancy of 0.3 (Fig. 1C and Supplementary Fig. S2). The cellobiose binds to subsites +1 and +2 making few interactions with the enzyme, which is consistent with the fact that cellobiose is the leaving group of the reaction and probably has low affinity for the enzyme. In molecules A and D, the glucose moiety bound in the +1 subsite interacts through its O3 and O4 hydroxyl groups with OE1 and OE2 of Glu216 (the acid/base catalytic residue). The O3 hydroxyl group interacts with the SD atom of Met 119 and the O2 hydroxyl group interacts with the main chain oxygen atom of Lys 135. The O6 hydroxyl group of the glucose moiety bound in the subsite +2 of molecule D interacts with the main chain oxygen and nitrogen of Lys 135. Trp121, Ile69 and Ile137 make hydrophobic interactions with the glucose moiety (Fig. 1D).

In molecule B which is not in complex with cellobiose, the residues 141–146 of the long β9/β10 loop are not defined in the electron density map whereas in molecules D and A, this region is stabilized by the interaction with cellobiose (Supplementary Fig. S1). The β9/β10 loop, close to positive subsites is very dynamic in the free enzyme and probably prevents crystallization of NopAAΔ48 in the absence of cellobiose. Analysis of the molecular structure in the asymmetric unit shows that it can undergo structural rearrangement upon cellobiose binding. This result suggests that this loop, displaying conformational stabilization after substrate binding, may play an important role in substrate recognition and specificity in the active site of NopAA. An equivalent loop, conserved in all clan-GH-C enzymes and referred to as ‘cord’, has already been identified in GH11 xylanases to undergo conformational changes upon substrate binding^27^.

### **NopAA from*Sinorhizobium fredii*USD257 has Xyloglucanase activity**

**Figure 2:**
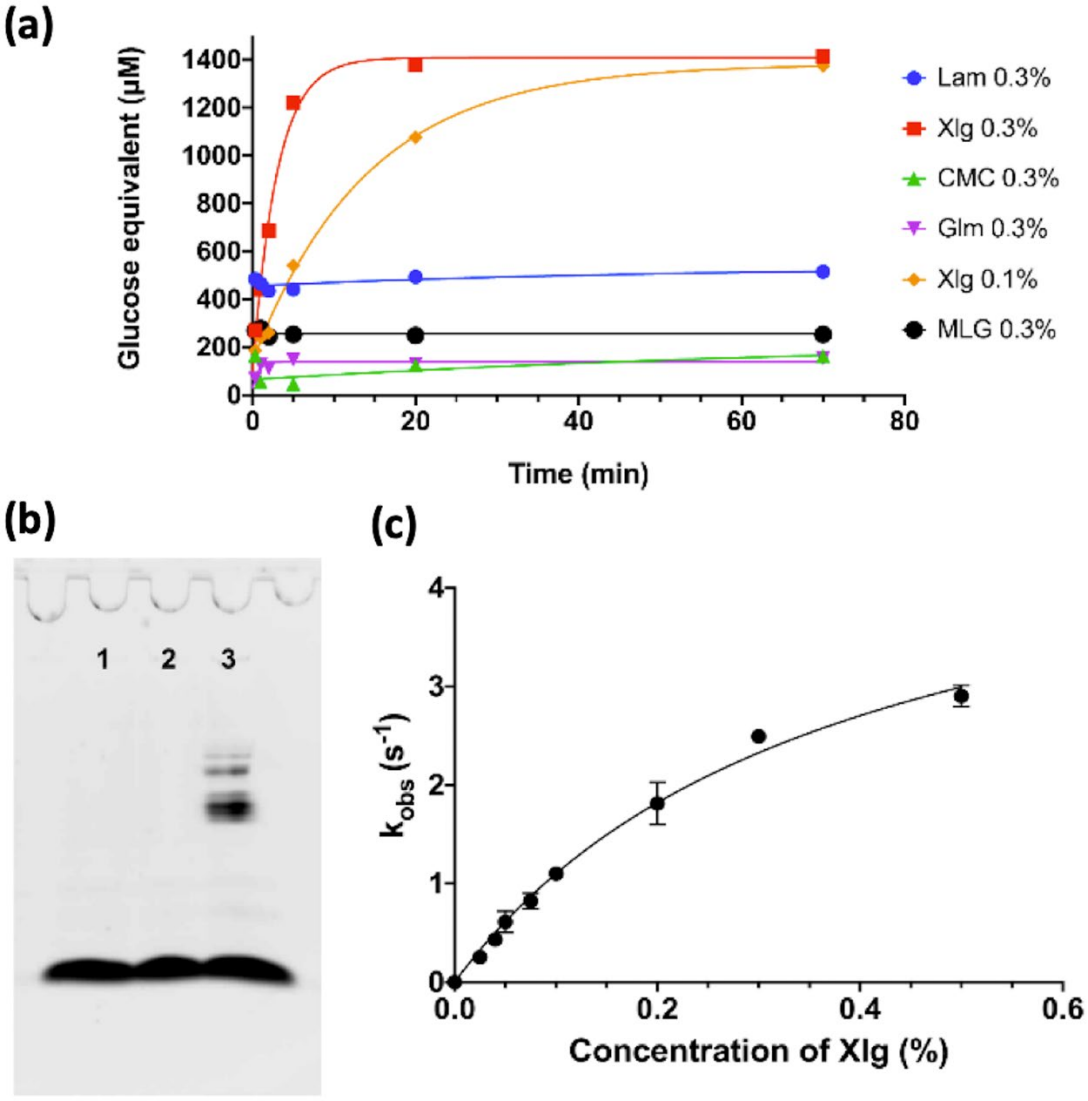
Enzymatic characterization of NopAA. (**a)** The activity of NopAAΔ48 was tested on different polysaccharides using the ferricyanide assay. (**b)** NopAAΔ48 was incubated with 0.5% of xyloglucan during 4 h. Lane 1, Xlg 0.5% without enzyme. Lane 2, Xlg 0.5% with boiled enzyme. Lane 3, Xlg 0.3% with 2.4 µM enzyme. Only the lane 3 displays a degradation pattern with oligossacharides of different length. (**c)** The kinetic parameters of NopAAΔ48 on xyloglucane were determined by fitting the data to the Michaelis-Menten equation. The errors bars represent s.d. from three replicate experiments. Even if the plateau is not completely reached, no higher concentration of xyloglucan could be tested because the viscosity of the mixture became the limiting parameter of the reaction.

NopAA analogs have been shown to have cellulase activity revealed with CMC-gelose assay combined with congo-red staining^22,23^. As NopAAΔ48 shows a cellulase activity analog to NopAA using this method (Supplementary Fig. S3) and the N-terminus 48 amino acid residues have been shown not to interfere with the catalytic domain of NopAA, we performed the enzymatic characterization of the enzyme using the catalytic domain (Fig. 2). In order to understand the physiological role of NopAA we decided to characterize its enzymatic activity. The ferricyanide reducing sugar assay was used to screen the hydrolytic activity of NopAA against several cellulose and hemi-cellulose substrates in six different buffers: sodium acetate pH 5.5 and 5.9, sodium phosphate pH 6.5 and 7.5, Tris pH 8, Glycine pH 8.6 with 150 mM NaCl. The best activity was found using the acetate buffer at pH 5.5 (data not shown), which is consistent with the fact that the glutamate nucleophile has to be deprotonated whereas the acid base catalytic glutamate has to be protonated. NopAA appears to be highly active exclusively on xyloglucan (Fig. 2a). Interestingly, the activity on CMC or β-glucan is only very faint or inexistent, in contrast to that observed for its homolog from *Bradyrhizobium japonicum*^22^. It is however detectable by the very sensitive test on CMC containing gelose (Supplementary Fig. S3). To confirm the detected activity on xyloglucan, we used fluorophore assisted carbohydrate electrophoresis (FACE). This electrophoretic technique allows visualizing small oligosaccharides, the degradation products of longer polysaccharide chains that do not enter the gel, which migrate as a function of their molecular weight and charge. The fluorophore used in this assay, 8-aminonaphthalene-1,3,6-trisulfonate (ANTS), is negatively charged and is used for both the migration property and the revelation of oligosaccharides of various sizes. In this assay, we can clearly see the bands that correspond to the degradation of xyloglucan into smaller oligosaccharides, as compared to both negative controls (Fig. 2b). Furthermore, NopAA seems to act as an *endo*-glycoside hydrolase as it generates long oligosaccharides, which are further degraded, whereas an *exo* enzyme would have generated a majority of small oligosaccharides of about the same size.

We also determined the kinetic parameters for NopAA on xyloglucan (Fig. 2c). NopAA displays a strong activity against xyloglucan, suggesting that NopAA might play a role in cell wall degradation. Moreover the *K*_M_ value of 0.38 (equivalent to about 10 µM considering an average M_W_ for the xyloglucan around 100 kDa^28^, is consistent with its putative role given that the concentration of xyloglucan in the cell wall is important.

### NopAA shows the structural basis for specific xyloglucan recognition

**Figure 3:**
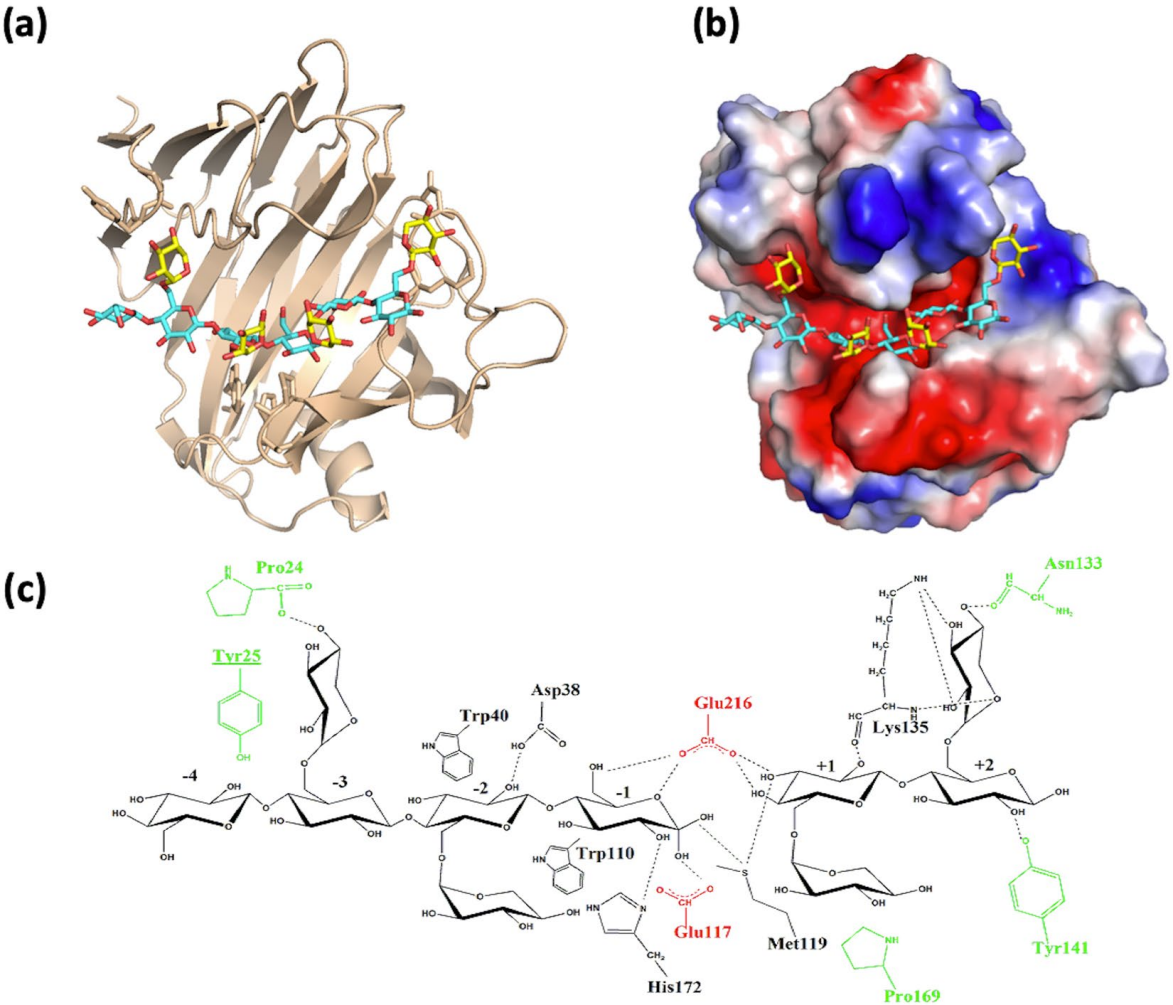
Structural representation of the substrate enzyme interaction in NopAA. (**a**) Overall structure of NopAA (chain D) in complex with xyloglucan. The protein is shown as a cartoon model and is colored beige. Xyloglucan substrate molecule is shown in balls and sticks, glucose moieties being colored in blue and xylose moieties in yellow. (**b**) NopAA in the same orientation as in panel A is represented in surface potential mode. Surface positively and negatively charged amino-acid residues are colored blue and red respectively. Xyloglucan substrate is represented in the same color code as in panel A, (**c**) schematic representation of NopAA/xyloglucan interaction. Catalytic residues of the enzyme are in red and residues interacting with xylose moieties which are not conserved in XEG and BlXG12 are in green.

To provide more insight onto the interaction of NopAA with xyloglucan substrate we simulated the complex between NopAA and the substrate of BlXG12 which possesses four xylose moieties attached to the glucose units in subsites −3, −2, +1 and +2^29^. This simulation revealed that NopAA can accommodate the xyloglucan in its active site (Fig. 3a,b). Superimposition of NopAA structure with the structure of the two known structures of xyloglucanases from GH12 in complex with xyloglucan, namely the xyloglucan-specific endo-β-1,4-glucanase (XEG) from *Aspergillus aculeatus* in complex (PDB ID code 3VL9^30^) and that from *Bacillus licheniformis* (BlXG12, PDB ID code 2JEN^29^) showed strong similarity within their active sites.

Comparing the global structure of these enzymes we see many differences in the insertion loops surrounding the active sites (Supplementary Fig. S4). NopAA possesses a specific insertion loop between β9 and β10 longer (25 residues, from 125 to 154) than the equivalent loops in the other xyloglucanases (10 amino-acid residues in XEG, and 4 residues in BlXG12). This loop lies close to subsites +1 and +2 of NopAA and is stabilized by interactions with the bound cellobiose. This loop has been shown to be dynamic and stabilized upon substrate binding in the active site of the enzyme (Fig. 1A) suggesting that it can adopt multiple conformations during the enzymatic process and may give NopAA enzymatic properties slightly different than XEG an BlXG12. In the model of NopAA in complex with xyloglucan, residues involved in the interaction with xylose moieties in subsites +1 and +2 belong to this loop. Lys 135 and Asp133 make hydrogen bonds with the xylose moiety of subsite +2 whereas Pro169 shares hydrophobic interactions with the xylose unit in subsite +1 (Fig. 3c).

On the contrary NopAA possesses two insertion loops, shorter than BlXG12 and XEG (Supplementary Fig. S4):

-the loop β8/β9 (residues 112–115) is 4 residues long in NopAA versus 12 amino acid residues in BlXG12 and 7 in XEG.

-the loop β6/α1 (residues 77–79) is 3 residues long in NopAA versus 15 for BlXG12 and 7 for XEG.

These two loops are located close to the subsite −2 of the enzymes. In the structure of BlXG12 they both interact directly with the bound substrate^29^.

The different lengths of these loops cause differences in the structure of the substrate binding site on the different enzymes. Thus, the presence of the β9/β10 loop of NopAA prolongs the binding site in the positive subsites while the presence of the loop β8/β9 in XEG and BlXG12 prolongs the substrate fixation site on the negative side (Supplementary Fig. S4).

**Figure 4:**
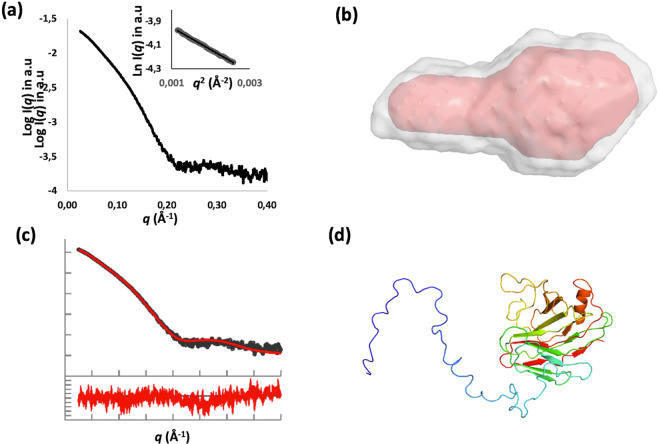
SAXS data and models for NopAA. (**a**) experimental SAXS data are plotted as function of the scattering vector q and Guinier plot is represented in the inset. (**b**) The average *ab initio* shape predicted by DAMMIF/DAMAVER for NopAA (white) superimposed with the most typical shape filtered by DAMFILT (red) (**c**) Theoretical profile of the best model of NopAA computed with AllosMod-FoXS (χ^2^ = 3.4) with residual plot shown below. (**d**) The best model of NopAA computed with AllosMod-FoXS shown as a cartoon model and coloured in rainbow from navy blue in the N-terminus to red at the C-terminus.

In subsite −3 of NopAA, Gln 212 replaces Glu201 in XEG and is in a good position to interact with the xylose O3 hydroxyl group. Asn36 replaces Tyr24 to interact with O5 of the xylose moiety, suggesting that NopAA is also able to accommodate a xylose moiety in subsite −3 even in the absence of aromatic residue. In XEG two tryptophan residues (Trp 13 and 28) involved in hydrophobic interactions with the glucose backbone are crucial for xyloglucanase activity. Trp 13 is replaced by Tyr 25 in the structure of NopAA, and lies in a good position to stack with a xylose residue (after a side chain flip). In the structure of BlXG12, there is no aromatic residue in this position, which is a xylose binding site. In these three enzymes it seems that subsite −3 can accommodate xyloglucan substrate in different ways, depending on the geometry and the shape of the enzymatic cleft of the enzymes. Comparing the surface of the three xyloglucanases, we see that the length of these two loops determines the shape of the catalytic groove of the enzymes and probably modifies their specificity and/or affinity for xyloglucan substrates (Supplementary Fig S4**)**. This result combined with enzymatic assays shows that NopAA has a xyloglucanase activity and is able to bind xylose moieties in its subsites. The dynamic properties of the long loop β9-β10 in the vicinity of positive subsites combined with the unfolded structure of the N-terminal domain (Fig. 4)) may help NopAA, to reach and bind xyloglucans localized in the extracellular of plant root cells during infection (Fig. 3c).

Although some Nops have been identified to interfere in certain symbiotic interaction, little is known about the role of T3E in the global nodulation process and further research is required to characterize their structural and biological properties. Symbiotic processes imply bacterial uptake into the plant cell. To this end, the bacterium has to break down and penetrate the plant cell wall, which is a strong physical barrier (for review^31^). However, the degradation has to be localized to avoid leakage of cellular content that could occur due to pressure of the vacuole against the cell wall. In this context, it is interesting to note that NopAA is specific to xyloglucan and not cellulose, the former being an important component crosslinking individual cellulose micro fibrils, but obviously not the most abundant cell wall component^32^. The hydrolytic activity of NopAA is thus coherent with helping symbiosis to be installed, without damaging entirely the plant cell protection.

### The N-terminal secretion signal of NopAA conserved structural features of pathogenic T3Es

Most T3Es possess a 48 amino-acids secretion domain, predicted disordered at their N-terminal end. The role of N-terminal domain of NopAA in translocation of the effector in eukaryotic cell has been demonstrated and this region can be recognized by a *Pseudomonas syringae* T3SS^19^. To study the structure of this domain and its position in the effector structure we used small angle X-ray scattering (SAXS), which gives information on quaternary structure of the proteins and, in our case, may allow to localize disordered domains of the proteins. NopAA shows smooth SAXS scattering profiles and the molecules are non-aggregated as shown by Guinier approximation analysis of the SAXS profile (Fig. 4a). For NopAA, the R_g_ (26 Å) and D_max_ (99 Å) were consistent with a rather monomeric globular structure prolonged by an elongated domain (Fig. 4a).

As expected the ten SAXS *ab initio* envelopes computed with DAMMIF^33^ (fitting the experimental data with χ^2^ between 2.35 and 2.54) showed that NopAA is organized in two domains (Fig. 4b):

-a globular domain fitting the C-terminal xylogulanase domain of the enzyme

-a disordered dynamic N-terminal domain emerging from the catalytic domain and that has been clearly identified as the secretion domain when compared with SAXS envelope obtained for NopAAΔ48 (Data not shown). The average model as well as the most typical model computed by DAMAVER are represented in Fig. 4b.

To go further in the description of the secretion domain, an atomic model of NopAA was computed using AllosMod-FoXS, a software combining small-angle x-ray scattering (SAXS) data and high-resolution protein structure^34^. The obtained model fits the SAXS data collected for NopAA with high degree of confidence(χ^2^ = 3.4) (Fig. 4c,d). This model, shows that NopAA is organized in a globular domain corresponding to the xyloglucanase C-terminal domain from which emerge the N-terminal secretion domain.

With respect to the catalytic cleft, SAXS results show that the secretion domain is located on the opposite side of the enzyme suggesting that it doesn’t interfere with substrate binding and enzymatic process.

The computed model proposes the secretion domain of NopAA to be an elongated peptide with few remaining secondary structure elements (Fig. 4d). This result shows for the first time a complete structure of a T3E before secretion comprising the disordered secretion domain emerging from the folded active C-terminal domain.

This structurally disordered conformation has been described for several T3E from animal-pathogenic bacteria^35^. It has been proposed that this partially folded region may give the effector the ability to bind specifically all the folded partners during pathogenic cycle from bacterial cytosol to eukaryotic target cell after secretion^1,36^. The secretion domain is probably involved in maintaining the xyloglucanase domain in good position to reach its substrates localized on plant roots cell wall. Its role after the translocation of NopAA into plant cells by T3SS is still unknown.

The partially folded structure of the N-terminal domain allows NopAA to recognize and bind secretion apparatus molecules with a mechanism that remains to be elucidated. Secretion of Nops depends on the presence of this signal that is interchangeable with secretion signal of T3E from pathogenic bacteria suggesting homology in substrate recognition by T3SSs. However, cognate chaperones, that have been shown to play a crucial role in substrate recognition and translocation in pathogenic bacteria, were not found in symbiosis island of *Rhizobium*.

In this work we showed that the secretion domain of NopAA is in an elongated conformation that can likely possess secondary structure elements as observed for the chaperone binding domains of T3E from pathogenic bacteria^35^. The elongated structure of secretion domain allows T3SS apparatus recognition in *Rhizobium* but also in *Pseudomonas syringae*^19^, a pathogenic bacteria in which several chaperones are used by T3SS for effector recognition^1^. At this stage, further investigations have to be performed to decipher the substrate recognition mechanism by symbiotic T3S apparatus.T3E may have conserved some structural features of a CBD that allow its direct recognition by T3SS apparatus. Another possibility has however to be considered as symbiotic T3E are spread into the genome, their localization is not restricted to symbiosis island and expression of these genes are flavonoid dependent and co-expressed with symbiotic *Nod* genes. At this step, although no chaperones have been identified in symbiotic island of rhizobia, it would be interesting to extend the analyse to check if one protein whose expression is flavonoid dependent can play this role.

## Methods

### Expression, purification and crystallization of NopAA and NopAAΔ48

The *Escherichia coli* strains *E. coli* one shot Top 10 (Invitrogen) and BL21(DE3) were used for construction of the plasmids and gene expression, respectively. All nucleotides and construction details are given in Supplementary Table S1. Sequence encoding for NopAA has been amplified from *Sinorhizobium fredii* USDA257 genomic DNA and cloned using the Gateway system in the destination vector pET-300Nt (Gateway) which allows the insertion of an N-terminal His6 tag on the construct. The resulting vector, pET-300Nt-NopAA was used as a template for the amplification of the sequence encoding NopAAΔ48 using the same protocol and leading to the destination vector pET-300Nt-NopAAΔ48. NopAAΔ48 corresponds to NopAA with a deletion of the first N-terminal 48 amino acids. pET-300Nt-NopAA and pET-300Nt-NopAAΔ48 were used to transform BL21(DE3) in order to produce protein samples for structural study. The same protocol was used for both constructions. Cells were grown at 37 °C in liquid LB broth supplemented with ampicillin (100 µg/ml) to an optical density of 0.6 (A_600_) and protein expression was induced during 3 hours at 37 °C with 0.5 mM IPTG.

Cells were collected, re-suspended in phosphate-buffered saline [PBS] buffer with 0.5 M NaCl, 0,01 mg/ml DNase, and complete protease inhibitor cocktail EDTA-free tablets [one tablet per liter of culture] (Roche) and were broken by two passages through a French pressure cell. Soluble bacterial extract was collected by centrifugation (12,000 × *g* for 1 hour at 4 °C).

The extract was subjected to a first step of Immobilized metal affinity chromatography using His-Trap HP 5 ml column (GE healthcare) equilibrated in PBS supplemented by NaCl 500 mM and Imidazole 20 mM. Recombinant protein was eluted by a pulse of Imidazole 250 mM in the equilibrium buffer. This step was followed by a size exclusion chromatography (SEC) step using a HiLoad 16/60 Superdex 75 (GE Healthcare Life Science). For SAXS experiments on NopAA the SEC column was pre-equilibrated with PBS supplemented with 150 mM NaCl and 10% glycerol. For crystallization trials the SEC column was pre-equilibrated with Sodium Acetate 100 mM pH 4.5, NaCl 150 mM, Glycerol 5% (w/v) for NopAAΔ48 and, NopAA.

For structural study, both protein samples were concentrated to 20 mg/ml by using Vivaspin centrifugal devices with a 5 kDa cut-off (Vivascience). Protein concentrations were determined with a NanoDrop 1000 Spectrophotometer (Thermo Scientific)

### Crystallization

Initial crystallization trials were performed in 96-well format using a 1:1 ratio of well solution to protein samples at 20mg ml-1 by screening at both 277 K and 293 K with commercial crystal screening kits. The protein solutions of NopAA 20 mg.ml^−1^, NopAAΔ48 20 mg ml^−1^ alone or supplemented with cellobiose powder (Merck) were submitted to crystallization trials. Crystal suitable for further experiments were obtained only for NopAAΔ48 in the presence of cellobiose at 293 K. Crystallization information is provided in Supplementary Table S2.

### Data collection and processing

For data collection NopAAΔ48/cellobiose crystals were flash-cooled in crystallization reservoir solution. X-ray diffraction experiment was conducted on the ESRF microfocus synchrotron beamline Id23-2 (Grenoble, France) at 100 K with a MarMOSAIC 225 mm CCD detector. All diffraction data were processed with XDS/XSCALE Package^37^. Statistics are shown in Table 1.

### Structure determination and refinement

The structure of NopAAΔ48 in complex with cellobiose was determined by molecular replacement with PHASER^38^ using a set of 8 superposed enzymes structures belonging to CAZY glycoside hydrolase family12 (GH12) as starting model (Chain A of PDB codes 1OA3^39^, 2JEM^29^, 2JEN^29^, 1OA4^39^, 1W2U^40^, 2NLR^41^, 4NPR (unpublished), 3VL9^30^, Supplementary Table S2).

The structure of the four molecules of NopAAΔ48 present in the asymmetric unit and the addition of glucose units was manually corrected using COOT^42^.The initial structure was refined with the program REFMAC5^43^ and PHENIX^44^. A set of 5% randomly selected reflections was set aside from refinement process to calculate the R_free_ factors throughout the refinement. The missing parts of the protein structure as well as water molecules were built, automatically with REFMAC-ARP/wARP. Refinement statistics are presented in Table 1. Polder map^45^ has been computed using PHENIX^44^.

### Enzymatic activity assays

Preliminary carboxymethylcellulose (CMC) gelose assay combined with congo-red staining were performed using the protocol described in^23^. To determine the specificity of NopAAΔ48, we monitored during one hour the degradation of the following substrates: carboxymethylcellulose 4 M (CMC), mixed-linked glucan (MLG, also referred to as β-glucan from barley, which contains a ratio of 1/4 β-1,3/β-1,4 linkages), tamarind xyloglucan (Xlg), glucomannan from konjac (Glm, which contains a ratio of 3/1 mannose/glucose units), and laminarin (Lam). All substrates were purchased from Megazyme, except for laminarin, which was purchased from Elicityl. The generation of reductive extremities due to the hydrolysis of the polysaccharide was monitored by ferricyanide assay described by Kidby and Davidson^46^. Unless otherwise stated, reactions were performed at 20 °C, using 180 µL substrate in acetate 30 mM, NaCl 150 mM pH 5.5 with 20 µL NopAAΔ48 (1.9 µM final). Aliquots of the reactions (20 µL) were taken at different times and added to 180 µL of ferricyanide reagent. The samples were then incubated for 15 min at 95 °C, cooled down to 20 °C. Then the absorbance was read at 420 nm using a Spark 10 M microplate reader (Tecan, Switzerland). As the hydrolysis of the substrates generates reductive glucoses, a calibration curve was performed under the same conditions using glucose solutions at concentrations from 0.1 to 1.2 mM as standard. The kinetic parameters were determined using a non-linear regression program (R program).

Fluorophore-assisted carbohydrate electrophoresis (FACE) was also performed to confirm the activity of NopAAΔ48 on xyloglucan. The fluorophore used in this assay, 8-aminonaphthalene-1,3,6-trisulfonate (ANTS) is negatively charged and is used for both the migration and the revelation of the poly/oligosaccharides. The reaction was carried out by adding 10 µL NopAAΔ48 (2.4 µM final) in 70 µL xyloglucan 0.5% in acetate 30 mM, NaCl 50 mM pH 5.5 during 4 h at 20 °C. The samples were heated to 90 °C to inactivate the enzyme and then dried under vacuum. They were resuspended with 2 µL of 150 mM 8-aminonaphthalene-1,3,6-trisulfonate (ANTS) and 2 µL NaBH_3_CN (1 M in DMSO) and incubated overnight at 37 °C. Next, 20 µL of glycerol 20% were added and 5 µL of the oligosaccharides were loaded in a 31% acrylamide gel. The electrophoresis was carried out at 4 °C, in the dark, during 2 hours at 175 V.

### SAXS data collection and analysis

Protein sample solution was centrifuged for 10 min at 10,000 rpm prior to X-ray analysis in order to eliminate all aggregates. SAXS experiments were conducted on the SWING beamline at Synchrotron SOLEIL (λ = 1.033 Å). The monodispersed samples of proteins were injected onto a size exclusion column (SEC-3, 150 Å; Agilent) using an Agilent HPLC system and eluted directly into the SAXS flowthrough capillary cell at a flow rate of 0.2 ml min^−1^ ^47^. Then 50 µl of protein samples were injected for SAXS measurements. The elution buffer consisted of PBS (pH 7.4), 150 mM NaCl, and 10% glycerol. Data reduction to absolute units, frame averaging, and subtraction were done using FOXTROT^47^. All subsequent data processing, analysis, and modelling steps were carried out with programs of the ATSAS suite^48^ (Fig. 4, Table 2). The radius of gyration was derived by the Guinier approximation using PRIMUS^49^ and the pair-distance distribution function was computed by GNOM^50^. Ten *ab initio* models were computed from the experimental data using the program DAMMIF^33^ and were averaged using DAMAVER^51^ and SUPCOMB^52^ to determine both common structural features and most typical shape of the protein.

**Table 2:** SAXS Structural parameters for NopAA.

Data collection Synchrotron source	SWING, SOLEIL
I(0) (cm^−1^)	0.024 ± 0.00004
R_g_ (Å)	25.93 ± 0.02
*q*_*min*_ (Å^−1^)	0.0012
*q*Rg max	1.21
Coefficient of correlation, R^2^	0.999
**P(r) analysis**	
I(0) (cm^−1^)	0.024 ± 0.00012
R_g_ (Å)	26.14 ± 0.02
D_max_ (Å)	99
*q* range _min_ (Å^−1^)	0.0310 to 0.5028
Porod volume (Å^3^)	44900
SASBDB code	SASDHG4

The program AllosMod-FoXS^34^ was used to model the disordered N-terminal secretion domain of NopAA from the X-ray crystallography structure of NopAAΔ48 and SAXS data of NopAA full length.

### Molecular modelling

Refined structure of NopAA was used as the target in order to model the putative binding poses of xyloglucane. A genetic algorithm was used for the search step within a sphere of 10 Å centred on the carboxylate moiety of the catalytic Glu216 residue. The scoring function was based on the ChemPLP forcefield, as used by GOLD^53^. Ligand was considered as very flexible and the other parameters were kept by default. A subsequent energy minimization was performed on the best model using the Amber forcefield.

All figures representing molecular structures of proteins and ligands were generated using PyMol (The PyMOL Molecular Graphics System, version 1.8.0.0 Schrödinger, LLC).

## Supplementary information

Supplementary Information.

## Data Availability

Structure have been deposited with the Protein Data Bank with the PDB ID code 6SDU. SAXS data have been deposited with the Small Angle Scattering Biological Data Bank (SASBDB) with the accession code SASDHG4.
