# Supplementary material for: Structural and enzymatic characterisation of the Type III effector NopAA (=GunA) from Sinorhizobium fredii USDA257 reveals a Xyloglucan hydrolase activity
Source: Sci Rep. 2020 Jun 18;10:9932. doi: 10.1038/s41598-020-67069-4 (PMC7303141; doi:10.1038/s41598-020-67069-4)
Supplement: Supplementary file 1 — Supplementary Information. [file 41598_2020_67069_MOESM1_ESM.pdf]

Structural and enzymatic characterisation of the Type III effector NopAA (=GunA) from *Sinorhizobium fredii* USDA257 reveals a Xyloglucan hydrolase activity.

Jonathan Dorival, Sonia Philys, Elisa Giuntini, Romain Brailly, Jérôme de Ruyck, Mirjam Czjzek, Emanuele Biondi & Coralie Bompard***

Supplementary information

Supplementary table S1: Macromolecules production information**.**

Supplementary table S2: Crystallization data.

Supplementary table S3: GH12 endoglucanases compared with NopAA in this study.

Supplementary Figure S1: Superposition of the structure of the four molecules of NopAAΔ48 in the asymmetric unit.

Supplementary Figure S2: Polder map of cellobiose in chain A contoured at 3.6 σ.

Supplementary Figure S3: Cellulase activity in a CMC Congo red agar assay.

Supplementary Figure S4: Comparison of the structure NopAA, BlXG12 and XEG.

Supplementary Table S1 : Macromolecules production information

Source organism	*Sinorhizobium fredii* USDA257
DNA source	Genomic DNA
Forward primers	CACCATGCCGATTTGGAGTTCGCACGC (NopAA)<br>CACCTCACTTCGAGGTCACAGACCAGT(NopAAΔ48)
Reverse primer	ATGGATTCCAATCGGATAAACGG
Cloning vector	pENTR/D-TOPO (invitrogen)
Cloning Host	E. coli one shot Top 10 (invitrogen)
Expression vector	pET300- Nt (invitrogen)
Expression Host	*E. coli* BL21 (DE3)
Complete amino-acid sequences of the constructs produced	
NopAA	<u>MHHHHHHITSLYKKAGM</u>DSNRINGGAAKGSPTGYVRTQEDHDLFRQAANEAGS<br>LSSAALVSARAPIWSSHAPYGSFSRDGYSWNNDVWGPRPGPQTISVSGVNRWS<br>VWSDQPNTPGIKSYPHVAFNIGKPLSSINTLSSSFNQEVPTGGAWDVAYDIWDSS<br>NKHEIMLWTNYTGNSDGSGNVKPISYHYAPSGAAIPVYSNVNVGGATWNVFEG<br>EGPDGHKVISLLRTSKTNSGTVDIKSILQWIKSKGYFGDIEVGSVQYGVEITSSPGGK<br>NFNFNNWSVTSK
NopAAΔ48	<u>MHHHHHHITSLYKKAGM</u>PIWSSHAPYGSFSRDGYSWNNDVWGPRPGPQTISVS<br>GVNRWSVWSDQPNTPGIKSYPHVAFNIGKPLSSINTLSSSFNQEVPTGGAWDVA<br>YDIWDSSNKHEIMLWTNYTGNSDGSGNVKPISYHYAPSGAAIPVYSNVNVGGA<br>WNVFEGEGPDGHKVISLLRTSKTNSGTVDIKSILQWIKSKGYFGDIEVGSVQYGVEI<br>TSSPGGKNFNFNNWSVTSK

Supplementary Table S2 : Crystallization data

Method	*Sitting drop vapour diffusion*
Temperature (K)	293
Protein concentration (mg. ml-1)	20.5
Buffer composition of protein solution	Sodium acetate 20mM pH 4.5, cellobiose 20mM
Composition of reservoir solution	4M Sodium formate, 0.1M Tris pH 8.5
Volume (µl) and ratio of drop	1 (1:1 protein : reservoir solution)
Volume of reservoir (µl)	200

**Supplementary Table S3** : **GH12 endoglucanases compared with NopAA in this study**		
GH12 endoglucanases (reference number)	PDB entry code	Amino-acid sequence identity with NopAA
Hydrolase from *Trichoderma citrinoviride*[39]	1OA3	33.5%
Xyloglucanase from *Bacillus licheniformis*[29]	2JEM	35.5%
Xyloglucanase from *Bacillus licheniformis* in complex with ligand[29]	2JEN	35.5%
Hydrolase from *Streptomyces lividans*[39]	1OA4	35.6%
Hydrolase from *Trichocladium griseum*[40]	1W2U	31.2%
Hydrolase from *Streptomyces lividansin complex with ligand*[41]	2NLR	32.4%
Xyloglucanase from *Aspergillus niveus* (unpublished)	4NPR	30.3%
Xyloglucanase from *Aspergillus aculeatus*[30]	3VL9	22.7%
Hydrolase from *Trichocladium griseum*[26]	1ORL	26%

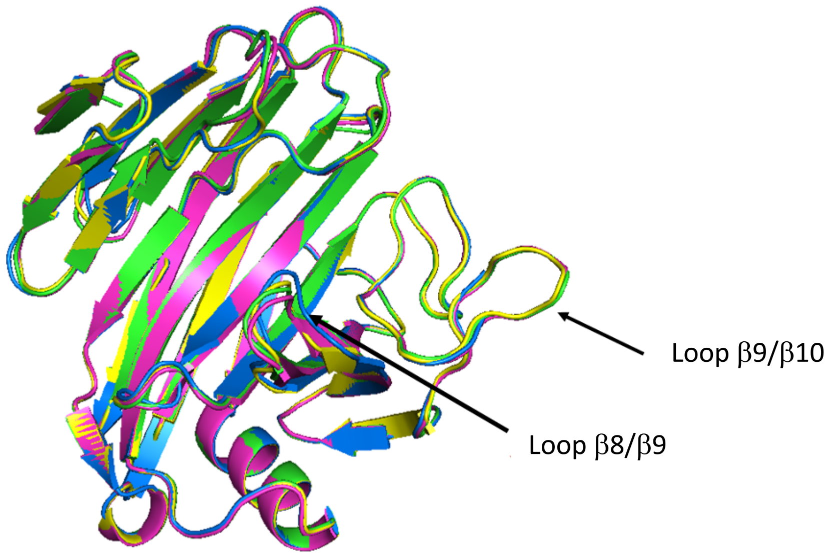

Supplementary Figure S1: superposition of the structure of the four molecules of NopAAΔ48 in the asymmetric unit. The protein molecules are shown in cartoon. Molecules A, B, C, and D are coloured green, blue, pink and yellow respectively.

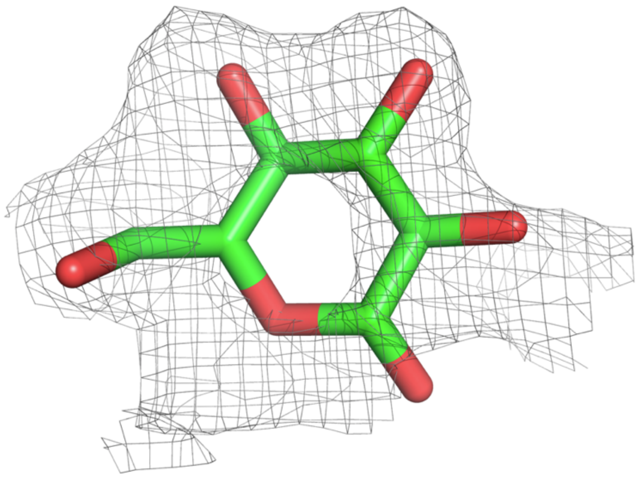

Supplementary Figure S2: Polder map of cellobiose in chain A contoured at 3.6 σ.

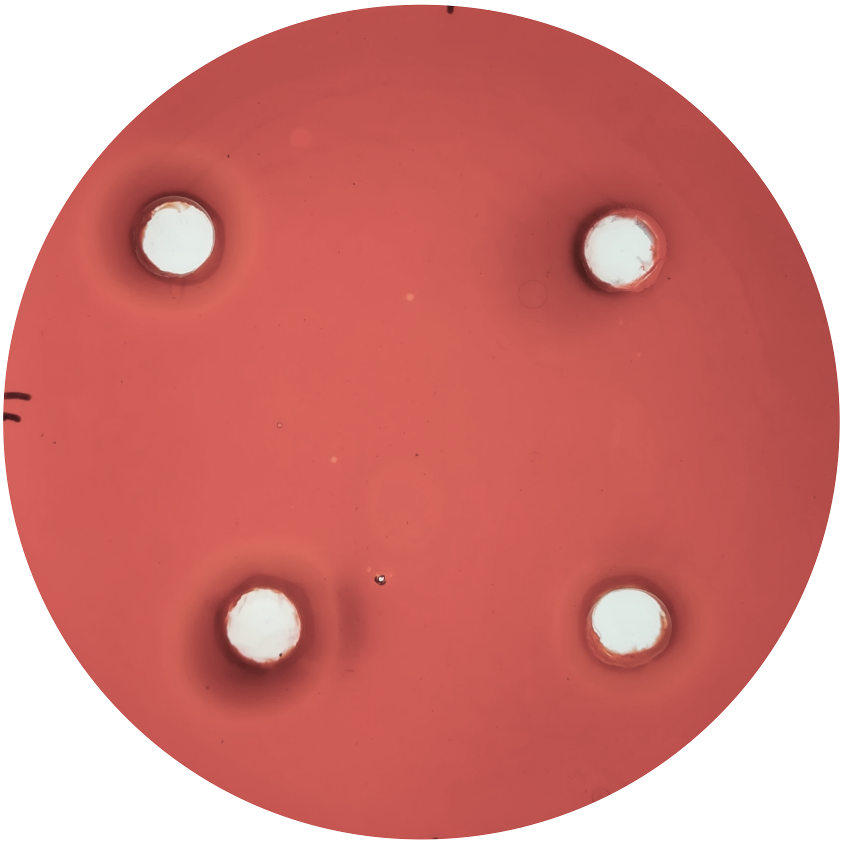

Supplementary Figure S3: Cellulase activity in a CMC Congo red agar assay. Hydrolysed CMC Cellulose appears as a diffusion zone around the wells where 30µl of NopAAΔ48 1mM (up left) 0.5 mM (down left) 0.3mM (down right) protein solutions have been applied respectively. 30µl of Sodium acetate 0.1M pH5 has been applied in the upper right wells as a negative control.

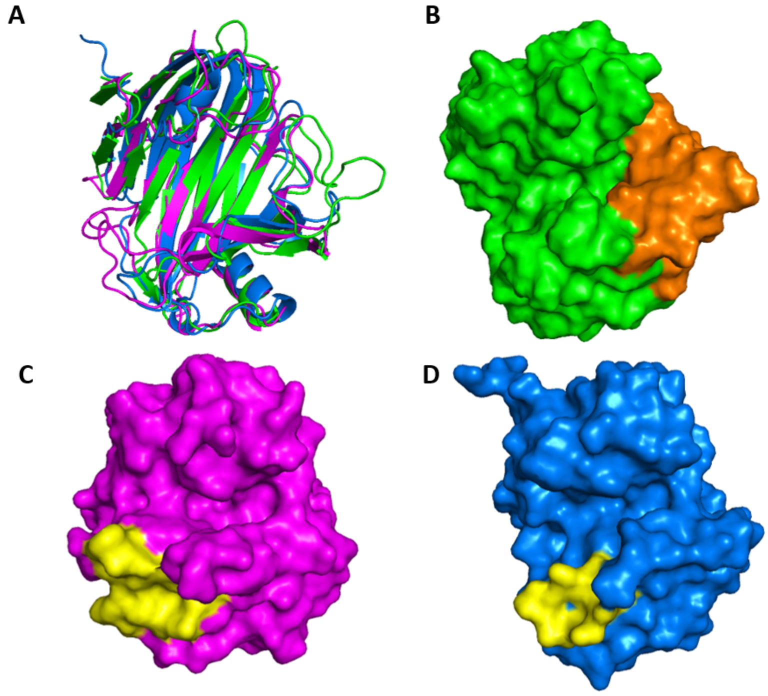

Supplementary Figure S4: comparison of the structure NopAA, BlXG12 and XEG. A) superposition of NopAA (green), BlXG12 (magenta) and XEG represented as cartoon. B) surface of NopAA in green, residues of the loop β9/β10 are coloured orange C) surface of BlXG12 in magenta D) surface of XEG in blue. In C and D residues of the loop β8/β9 are coloured yellow
